# Spin-Resolved Electronic and Transport Properties of Graphyne-Based Nanojunctions with Different N-Substituting Positions

**DOI:** 10.1186/s11671-019-3133-5

**Published:** 2019-08-28

**Authors:** Xiaobo Li, Yun Li, Xiaojiao Zhang, Mengqiu Long, Guanghui Zhou

**Affiliations:** 1 Hunan Key Laboratory of Super-Microstructure and Ultrafast Process, School of Physics and Electronics, Central South University, Changsha, 410083 China; 2 Physical Science and Technology College, Yichun University, Yichun, 336000 China; 3 Institute of Low-Dimensional Quantum Materials and Devices, School of Physical Science and Technology, Xinjiang University, Urumqi, 830046 China; 4 Department of Physics and Key Laboratory for Low-Dimensional Structures and Quantum Manipulation (Ministry of Education), Hunan Normal University, Changsha, 410081 China

**Keywords:** Molecular junction, γ-graphyne nanoribbon, N-substituting position, Spin-charge transport, First-principles calculation

## Abstract

Since the rapid development of theoretical progress on the two-dimensional graphyne nanoribbons and nanojunctions, here we investigate the electronic band structures and transport properties for the junctions based on armchair-edged γ-graphyne nanoribbons (AγGYNRs) with asymmetrically nitrogen (N)-substituting in the central carbon hexagon. By employing first-principles calculation, our computational results imply that the number and the location of single or double N-doping can efficiently modulate the electronic energy band, and the N-doping hexagonal rings in the middle of the junction play a vital role in the charge transport. In specific, the effect of negative difference resistance (NDR) is observed, in which possesses the biggest peak to valley ratio reaching up to 36.8. Interestingly, the N-doped junction with longer molecular chain in the central scattering region can induce a more obvious NDR behavior. The explanation of the mechanism in the microscopic level has suggested that the asymmetrically N-doped junction by introducing a longer molecular chain can produce a more notable pulse-like current-voltage dependence due to the presence of a transporting channel within the bias window under a higher bias voltage. In addition, when the spin injection is considered, an intriguing rectifying effect in combination with NDR is available, which is expected to be applied in future spintronic devices.

## Introduction

Several two-dimensional (2D) carbon materials have been demonstrated as the potential candidate for spintronic devices [1–5]. Recently, more and more experimental studies on 2D carbon materials have been performed on this aspect [6–11]. Particularly, the graphene [12–15] and graphyne [16–19] nanostructures and the related devices [20–22] have been proposed theoretically. Subsequently, the valuable effects of rectifying [12, 20], switching [13, 23], negative difference resistance (NDR) [23–25], and spin-filtering [26–28] have been observed in these devices. Further, the graphene and graphyne materials are considered to be the electrode materials of spintronic molecular junctions, because of their outstanding electronic and transport properties.

As we know, research works show that the graphene nanoribbons can be tailored and cut into many structures as molecular devices in experiment [29, 30]. Similarly, the graphyne structures [17–19, 31, 32] are made of carbon atoms, which hold adjustable electronic and transport properties better than that of graphene. Recently, the graphdiyne films have been demonstrated to generate on the copper surface by employing a methodology of cross-coupling reaction [8]. A rational approach to synthesize graphdiyne nanowalls by using a modified Glaser-Hay coupling reaction has been reported by Zhou et al. [9]. However, an interrelated experimental observation also remains a real challenge for a long time. Over time, the graphyne nanoribbon is also eager to be prepared into junctions in an experiment by employing the cross-coupling reaction method, energetic electron irradiation inside a transmission electron microscope [8, 29, 30]. Further, because of the inclusion of high carrier mobility and ongoing electronic characteristics [4, 33], the graphyne structures including α- [34, 35], β- [36], γ- [37], 6,6,12- [27], α-2- [38], δ- [39], 14,14,14-graphyne [40], and relative heterojunctions [41, 42] are getting more and more attention in theory. However, there is a lack in the investigations on the transport characteristics of several length-controlled molecular chains composed of repeated molecular units between two semi-infinite γ-graphyne nanoelectrodes.

The γ-graphyne nanoribbon (γGYNR) [43], which can be classified into armchair and zigzag edges, exhibits the semiconducting behavior with a band gap regardless of edges [18]. Furthermore, the armchair γGYNR (AγGYNR) is less used to construct a spintronic and molecular junction than the zigzag one [44–46], because it holds a larger band gap than the zigzag nanoribbon [18]. But the N-doping has been reported to change the electronic and transport properties of graphene and graphyne [47–51], which is capable of leading to narrowing the band gap. In an experiment, the N-doping has been implemented in the graphene sheet [52, 53]. However, the γGYNR has been predicted to be semiconductors exhibiting small carrier effective masses and high carrier mobility like graphene [4]. Previous theoretical researches about dopant have also displayed intriguing electronic or transport properties of GYNR [49, 50, 54, 55]. Previous experimental investigations on the graphdiyne NRs [8, 9] and device without or with N-doping [56, 57] have also been reported recently. Besides, the acetylenic linkages between two carbon hexagons for γGYNR provide much natural holes to realize the doping of various candidates as *n*-doping or *p*-doping semiconductors. Thus, it is essential to consider single or double N-doping in our proposed junctions of AγGYNRs here.

Motived to deeply understand the spin electronic and transport properties of several length-adjustable molecular chain sandwiched between two semi-infinite AγGYNRs with different N-substituting positions, we have finished the computational work by using first-principles calculation in combination with a Landauer-Büttiker approach in this paper. The results of theoretical simulation suggest that the N-doping can efficiently reduce the energy gap of 3-AγGYNR junctions, then the single N-doping of M_2_ and double N-doping of M_6_ can induce the spin splitting of energy band. The transport current of 3-AγGYNR junction without N-doping is weakened as the number of repeated units in the scattering region increases; in contrast, the currents are intensified with a longer molecular chain for 3-AγGYNR junctions with single or double N-substituting positions. Interestingly, the rectification and obvious NDR effects are observed in the N-doping junctions of M_2_ and M_6_. Such behaviors generate from the different coupling between two electrodes and the scattering area. In order to explain the mechanism of NDR behavior in a microscopic level, the reason displayed that the longer molecular chain contained in the asymmetrically N-doped junctions can induce a more obvious pulse-like current-voltage dependence due to the existence of an opened transporting channel within the corresponding bias window under higher bias. Additionally, the hexagonal ring with N-substituting positions has a vital impact in the transport process.

The paper is divided as follows: In the “Modeling and Computational Methods” section, the junction description and method are proposed. Next, we describe the results and discussions on their internal mechanisms in the “Results and Discussions” section, and the computational results are summarized in the “Conclusions” section.

## Modeling and Computational Methods

**Fig. 1:**
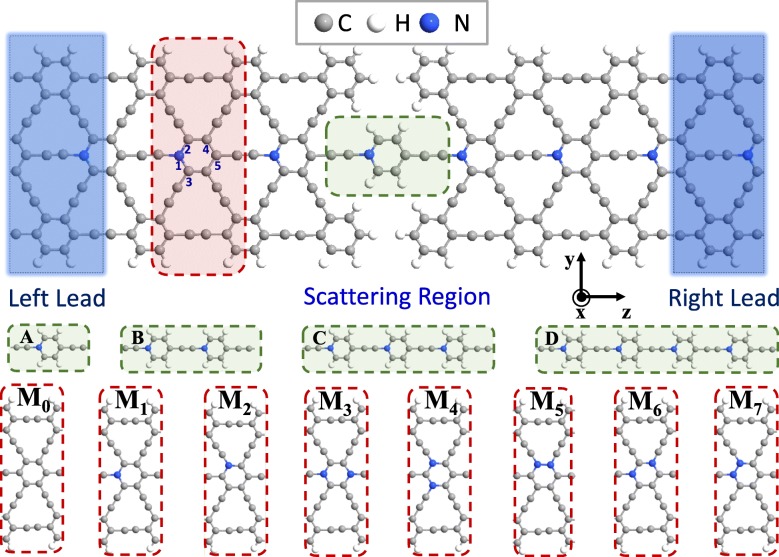
(Color online) Schematic views of the two-probe systems. For clarity, the device with single N-doping (the second super cell at the bottom panel as M_1_) of AγGYNR is chosen as the main diagram in the top panel. The blue shaded rectangular regions indicate the left and right leads, between which is the central scattering region. The gray, white, and blue solid spheres represent the carbon, hydrogen, and nitrogen atoms, respectively. The transport direction is along the *z* axis. Further, the green shaded molecular unit at the main diagram can be periodically replicated leading to produce the four structures with different lengths of molecular chains including benzene and acetenyl molecules in the middle panel, which have been named as A–D. In addition, the red shaded frame denotes the periodic super unit cell of the nanoribbon which can be changed by the ones of M_0_–M_7_ without or with single/double N-substituting positions in the bottom panel. For convenience, the atomic positions of C_6_ ring are numbered as 1, 2, 3, 4, and 5 as pointed under the corresponding atoms of the red frame, respectively

The molecular wires consisting of 1~4 repeated molecular units, which are made of one benzene and one acetenyl without or with N-doping, are shown in the middle panel of Fig. 1 with four green dashed rectangular boxes. The scattering region of molecular chain with N-substituting position is sandwiched between two symmetric semi-infinite AγGYNRs, where 1-repeated molecular chain (A), 2-repeated molecular wire (B), 3-repeated molecular chain (C), and 4-repeated molecular chain (D) are applied, respectively. We choose the 3-AγGYNRs as the electrode here due to the symmetric structure of a π-σ-π architecture. The left lead, scattering region, and right lead are contained in our designed nanojunctions, and all the carbon atoms at the edge of devices are saturated by the hydrogen atoms to improve the stability of structures [18, 43, 45, 46]. For our proposal devices, the molecular chains are convenient to be trailed or sculptured directly into junctions by a mechanical method or chemical reaction from a whole γGYNRs in experiment like the other structures [29, 30, 56]. For clarity, the main view in the top panel of Fig. 1 is employed by the super unit cell with single N-substituting position in the central position, which is named as M_1_ in the second picture of the bottom panel in Fig. 1. For convenience, the atomic substituting positions of C_6_ ring are numbered as 1, 2, 3, 4, and 5 as pointed under the corresponding atoms of the red frame, respectively. Similarly, the pristine device without N-doping is called as M_0_, where the models with two-typical single N-substituting positions (replacing the atomic positions of 1 or 2) are M_1_ and M_2_, and the ones with five-typical double N-doping at different substituting positions (replacing the atomic positions of 1/5, 2/3, 2/4, 1/4, and 1/2) are named as M_3_–M_7_, respectively. The red shaded part enclosed by a dashed rectangular box in the main view of Fig. 1 is the periodic super cell of the nanoribbon, which is replaced by the eight models. Therefore, there are 32 typical models which have been researched in total. For instance, the junction of M_1_ with the single N-instituting position of 1 including a molecular chain of four repeated molecular units as D should be call for M_1D_.

We firstly optimize all the structures of unit cells and molecular junctions by implementing the density functional theory calculation in the Atomistix ToolKit (ATK) package [46–48, 58] According to the results of optimization, the bond distance of the nitrogen and carbon atoms approaches 1.43 Å, which is suitable to replace the carbon atom with a similar bond length 1.43~1.46 Å of a C–C bond in γGYNRs [31, 59]. Moreover, the C ≡ C bond of system between the nearest neighbor benzenes is still stable after the optimization. We choose the structures as our models with lower total energies. The energy difference between super unit cells with single N-doping is 0.57 eV, and the one with double N-doping increases up to 1.63 eV, which is thought to be easier to realize experimentally. So, these molecular junctions can be applied as new devices with N-doping. The detailed computational parameters have been implemented as follows. We use norm-conserving pseudopotentials and the spin-generalized gradient approximation with Perdew, Burke, and Ernzerhof functional for exchange-correlation potential [60–62]. The computational geometries are optimized until all residual forces on each atom are smaller than 0.02 eV Å^−1^. To perform the calculations of electronic structure, a k-point grid of 1 × 1 × 15 Monkhorst-Pack in Brillouin zone is adopted. The Monkhorst-Pack mesh of reciprocal space sampling for the spin-dependent transport calculation is 1, 1, and 100 in the *x*, *y*, and *z* directions, respectively, and the cut-off energy is adopted to 150 Ry. The double-*ζ* polarized basis is set to all elements including C, H, and N. Furthermore, the convergence criterion for total energy is set to 10^−5^ eV. Since the transport direction is set to the *z* axis, an interlayer vacuum distance of ~ 25 Å is used to avoid interactions between the periodic images [63, 64]. The transmission spectrum as a function of energy (*E*) and bias voltage (*V*) is defined as

$$ T\left(E,V\right)=\mathrm{Tr}\left[{\Gamma}_L\left(E,V\right){G}^R\left(E,V\right){\Gamma}_R\left(E,V\right){G}^A\left(E,V\right)\right], $$

where *G*^*R*(*A*)^ is the retarded (advanced) Green’s function of the central scattering area and Г_*L*(*R*)_ is the coupling matrix of the left (right) electrode. The spin transport current is calculated by using the Landauer-Büttiker formula [65–68]

$$ I(V)=\left(\frac{\mathrm{e}}{h}\right){\int}_{\mu_L}^{\mu_R}T\left(E,\right.\left.V\right)\left[{f}_L\left(E-{\mu}_L\right)-{f}_R\left(E-{\mu}_L\right)\right] dE, $$

where the *μ*_*L*/*R*_ = *E*_F_ ± *eV*/2 is the electrochemical potential in terms of the Fermi energy (*E*_F_) of the material common to both leads under an external *V*, and the Fermi-Dirac distribution function is ${f}_{L/R}(E)=1/\left[1+{e}^{\left(E-{\mu}_{L/R}\right)/{\kappa}_BT}\right]$ in the left/right lead.

## Results and Discussions

**Fig. 2:**
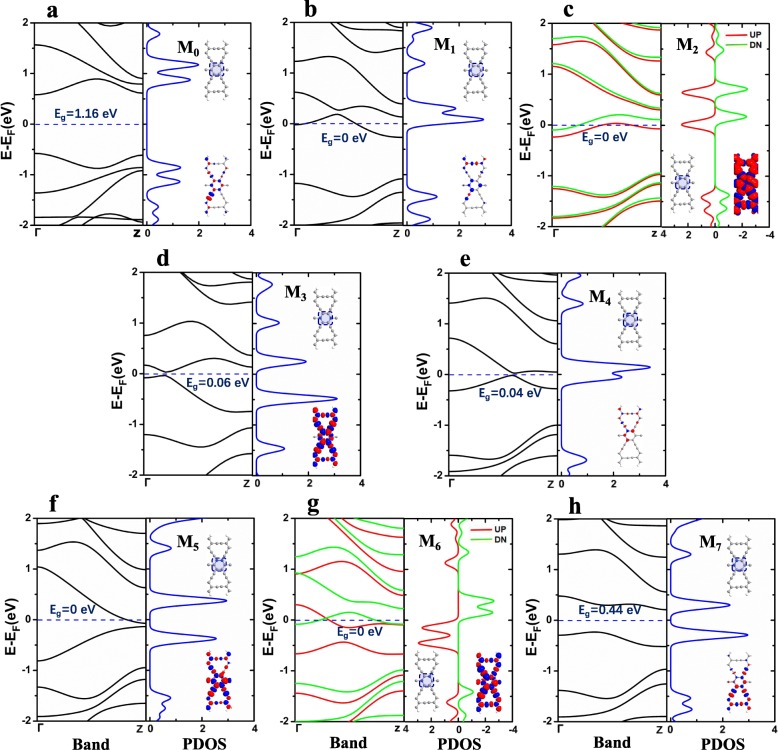
(Color online) The electronic band structure (in the left panels) and spin density distribution (the insets in the right panels of each pictures with red and blue clouds which indicate positive and negative electrons) for the super unit cells of M_0_–M_7_ corresponding to the **a**–**h**. The horizontal blue dashed line is also drawn to indicate the position of the Fermi energy level. The projected density of state (PDOS) in the right panels of **a**–**h** is the density of state with respect to all the atoms of the hexagonal rings inside the blue shaded frame, respectively. Here, the red and green lines represent the spin-up (UP) and spin-down (DN) components for M_2_ and M_6_ in **c** and **g**

To perform the practical electronic band structure calculations, the periodic super unit cell with N-doping along the *z* direction of the ribbon axis is considered. For the convenience of contrast observation, we show all the unit cells in the form of illustrations for M_0_–M_7_ in Fig. 2a–h. For our proposed junctions, the central hexagonal ring containing the N-instituting position is considered to play a significant influence in the transport properties. Therefore, the central C_6_ rings with N-doping are enclosed in a blue dashed frame with a blue shaded area, in which the projected density of state has also been calculated and shown in the right panels of Fig. 2a–h.

Firstly, we investigate the structural and electronic characteristics of AγGYNRs. As shown in Fig. 2a, the electronic band of pristine super cell for M_0_ displays that the AγGYNR is a semiconductor with a direct energy gap of 1.16 eV. The lowest conduction band and the highest valence band originate from π* and π sub-band, respectively [37, 69]. But for M_1_ and M_2_ with single N-instituting position in Fig. 2b and c, an obvious impurity band stretch across the Fermi level, leading to producing zero energy gap. Interestingly, the electronic band structure of M_2_ is spin splitting. The inclusion of single N-doping narrows the energy gap at the Brillouin zone boundaries. As a result, the band structures for M_1_ and M_2_ behave metal property. When the unit cell of system doped with double N-doping for M_3_–M_7_ in Figs. 2d–h, some new properties of the band structures have been discovered. The energy gaps of M_3_, M_4_, and M_7_ have been narrowed into 0.06, 0.04, and 0.44 eV due to the using of dopant in the pristine structure, which images that they are still semiconductors after double N-doping. However, we can find that the band structures of M_5_ and M_6_ perform metallicity with zero band gap in Fig. 2f and g, resulting in that it is of importance for the transport behavior. Similarly, the spin splitting of the electronic band structure arises in the double-doped structure of M_6_ in Fig. 2g. Note that the appearing of metallicity depends on the typical N-instituting positions in the central C_6_ ring of AγGYNR. As shown later, the central part of the C_6_ ring indeed influences the conduction properties of AγGYNRs reported in our present work.

To deeply illustrate the impact of N-instituting position, the spin-dependent electrons on N atoms can be identified from the spin density distribution of the AγGYNRs (see each inset in Fig. 2a–h). As displayed in Fig. 2c and g, obviously, the spin-dependent scattering of electrons is increased owing to the introduction of single or double N atoms; as a result, the magnetism of the AγGYNRs is enhanced compared with the pristine one in Fig. 2a. Meanwhile, the relative rich hopping and scattering of electrons can also be found in Fig. 2d and f. For those four pictures of spin densities, the distributions of spin-dependent electrons have been spread to all the unit cells, leading to the consequence that it contributes to the charge transport. Nevertheless, the distributions of the electron density are partly localized in the central part of the insets for Fig. 2b and e, whereas for Fig. 2h, it is localized in the central and bottom part of the inset. Thus, the dopant in the central hexagonal ring of super cells plays a main impact in the electronic band. Our observation is transferred to the region of C_6_ ring in our proposal structure.

In addition, the eight models have been shown as insets in the right panel of Fig. 2a–h, where the hexagonal rings with N-substituting positions are enclosed with the blue shaded dashed frames in the model, respectively. The PDOS of the hexagonal rings are plotted in the right panel of Fig. 2a–h. The results suggest that the PDOS of the designated area in M_0_–M_7_ can match the corresponding electronic band structures well; especially, the π* and π sub-bands near to the Fermi level mainly originate from the contribution of the six-membered ring. For the original model of M_0_ in Fig. 2a, there is no peak of PDOS around the *E*_F_ leading to a wide energy gap, which results in a semiconducting property. If the typical C atoms in the C_6_ ring are replaced by single or double N atoms as M_1_–M_7_, the double peaks of PDOS trend to move close to the *E*_F_ contributing to the decrease of a band gap. For instance, there are two high peaks of PDOS around the Fermi level (see Fig. 2b and e) for M_1_ and M_4_; to a great extent, they contribute to narrow the band gap at the first Brillouin zone. More interestingly, the spin-up and spin-down energy bands for M_2_ and M_6_ (see Fig. 2c and g) are splitting as a result from that the spin-up (spin-down) PDOS move down (up) to a lower (higher) energy state. However, for M_3_, M_5_, and M_7_ in the right panels of Fig. 2d, f, and h, there also exist two separate peaks of PDOS near the Fermi level, which contributes to the appearing of π* and π sub-bands. Therefore, the N-doping in the central C_6_ ring part of M_0_–M_7_ is a vital issue, and it is interesting to continue to study the electron transport of AγGYNRs designing from the eight original super cells.

**Fig. 3:**
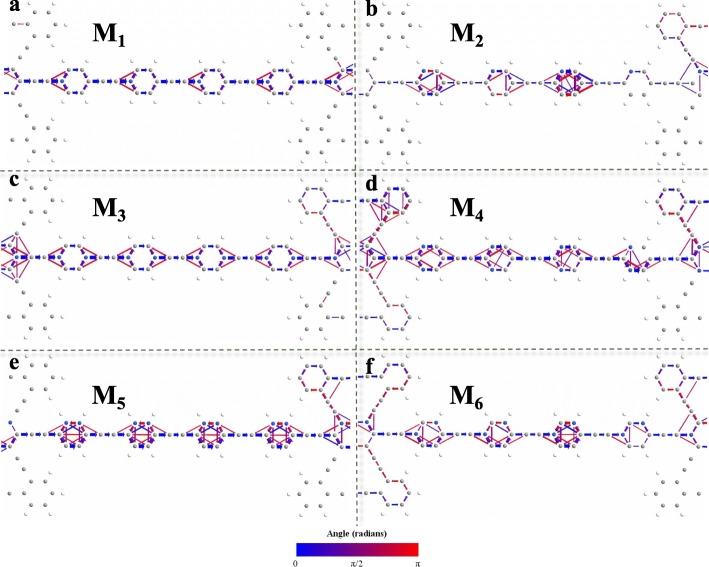
(Color online) The transmission pathways of N-doping AγGYNRs as M_1_–M_6_ with four periodic molecular chain named as D in the central region. In the views of **a**–**f**, the colors of connecting arrows between two atoms give the hopping direction of electron transmission according to the drawn color bar, and the successive different colors correspond to a series of different angles. The threshold is taken as 0.05

In order to illustrate the transport properties of AγGYNRs, we plot the transmission pathways of N-doping AγGYNRs to display the transmission probabilities of nanoribbons in Fig. 3. Omitting the pictures with terribly small distributions of transmission pathways for M_0_ and M_7_, the devices M_1_–M_6_ including the molecular chains with four repeated unit cells named as D in the central scattering region are considered. For M_0_ and M_7_, the transmission pathways are broken with no transport channel, and the hopping and scattering of electrons only appear in the left electrode, so their distributions of transmission pathways are ignored here. All the six devices display a perfect transport channel in Fig. 3a–f, which image that the electrons can flow from the left lead to the right one. In fact, the electrons can go through the central scattering area resulting from the inclusion of N-doping. As displayed in Fig. 3a and b for M_1_ and M_2_, the electronic transition does not only take place between the nearest neighbor atoms but also between the next nearest neighbor atoms. Similarly, when the double N-doping is applied for M_3_–M_6_ in Fig. 3c–f, more rich electronic transition happens to the next nearest neighbor atoms.

**Fig. 4:**
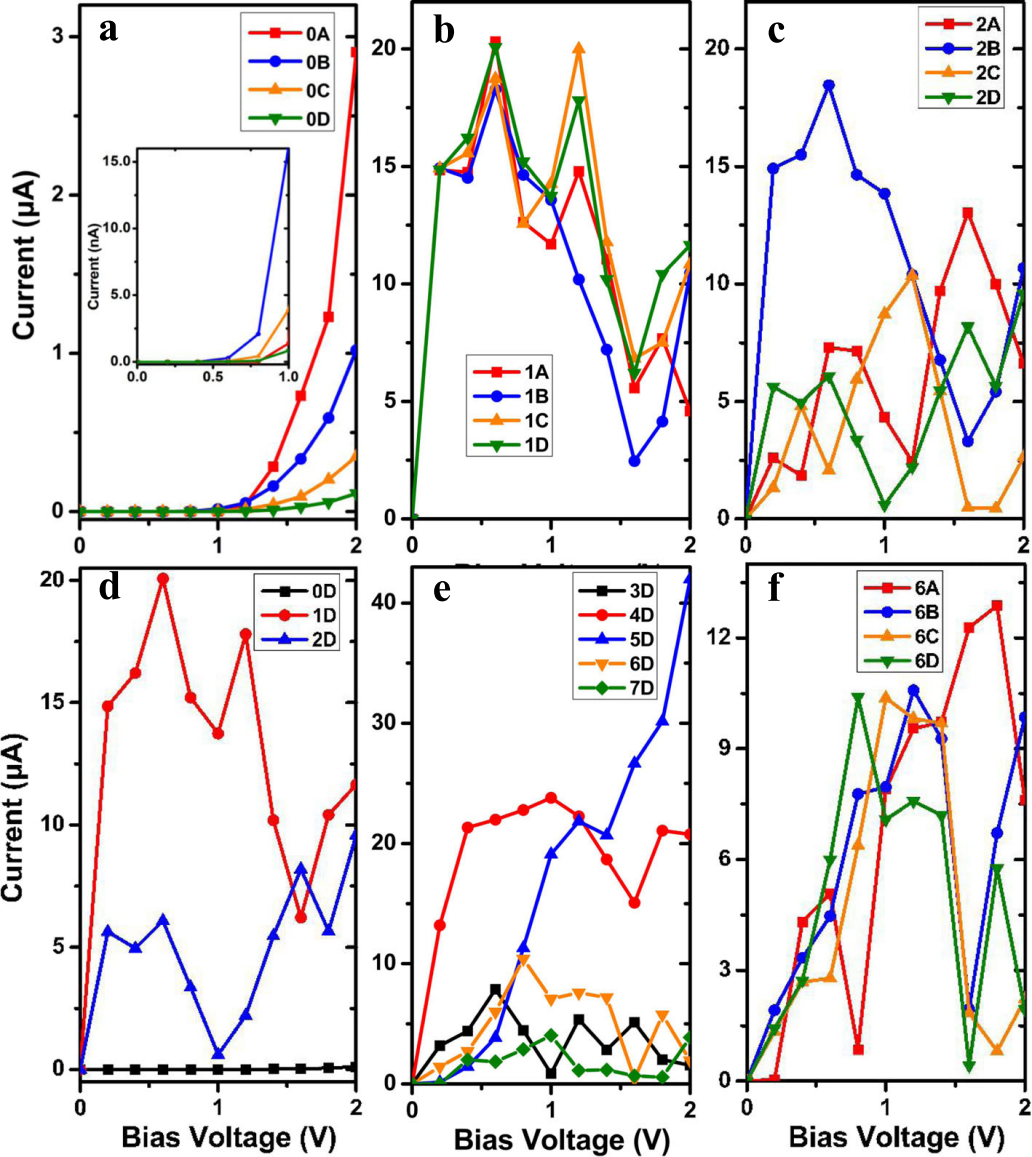
(Color online) The current-voltage (*I-V*) characteristics of AγGYNRs (**a**) without N-doping or with single N-doping as shown in **b** M_1_ and **c** M_2_ for the four different molecular chains as A–D. The *I-V* curve of AγGYNRs with the four periodic molecular chain as D for **d** M_0D_–M_2D_ or **e** M_3D_–M_7D_. **f** The *I-V* curve of AγGYNRs for the four different molecular chains as A–D for M_6_

Further, we continue to focus on the central scattering region of molecular chains, finding that the next nearest electronic transition is used to take place around the N atoms for all displayed models in Fig. 3. So, the N-doping plays an important action on the electronic transition, which contributes to producing a stronger current in Fig. 4. More interesting, most of the transmission pathways localize in the C_6_ rings of AγGYNRs, indicating that N-doped C_6_ rings track a main contribution for these nanojunctions. In the left column of Fig. 3 for M_1_, M_3_, and M_5_, the transmission pathways exhibit a symmetric distribution during the molecular chains. But for M_2_, M_4_, and M_6_ in the right column, they behave weaker electronic transition trends in the fourth molecule of the scattering region as shown in Fig. 3b, d, and f. Thus, a longer molecular chain above four repeated super units is not suitable to perform in these typical junctions. Especially, the pathways of electronic transition for M_5_ in Fig. 3e distribute more possibilities of transport channels than the other ones. The back-scattering of electrons trends to be enhanced at the upper edge of molecular chains due to the existence of double N-doping atoms for M_5_ and M_6_ in Fig. 3e and f. Consequently, the N-dopant brings into play the vital influence in the charge transport of AγGYNR junctions. Additionally, the asymmetric distributions of transmission pathways for M_2_ and M_6_ in Fig. 3b and f are possible to display some ongoing physical behaviors. The corresponding discussion is of interest to be continuously exhibited. Next, we want to show the current curves for these junctions to find more interesting phenomena.

**Fig. 5:**
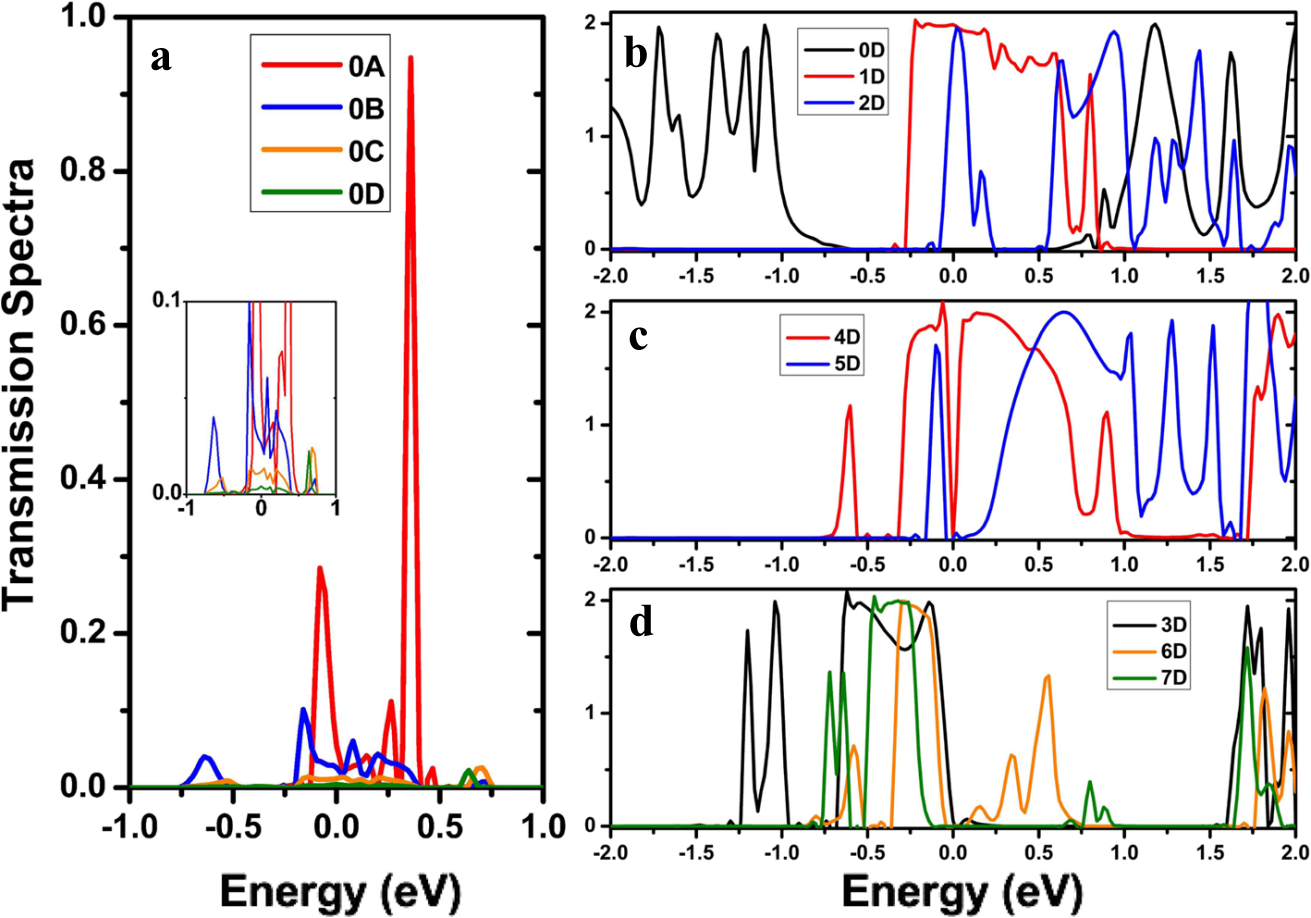
(Color online) The calculated transmission spectra of AγGYNRs at zero bias **a** without or **b**–**d** with various N-doping models in the horizontal molecular nanowires, where the corresponding repeated unit cell is displayed at Fig. 1. **a** The transmission spectra for non-doping AγGYNRs for M_0_ including four molecular chains with different molecular lengths for A–D; the color of solid lines in the figure is consistent with that in Fig. 2a. The inset is the partly blow-up view of the main view where the transmission spectrum is less than 0.1. Similarly, the solid lines in **b** are consistent with those lines with common colors in Fig. 2d for M_0D_–M_2D_, and the solid lines in **c**/**d** correspond to the ones in Fig. 2e for M_3D_–M_7D_, respectively

To further understand the transport properties of these two-probe junctions, we compute the *I-V* curves for AγGYNR junctions with four different molecular chains of different lengths in Fig. 4. As we focused our work on the produced structures of N-instituting positions, the effect on the length of molecular chains on structure-dependent transport properties has not been explicitly considered. The pristine device for M_0_ has been investigated in Fig. 4a. There is a threshold voltage of ~ 1.2 V, below which the conductance gap increases with the increasing of bias voltage, resulting from the shifting of band structures (see Fig. 2a) in the left and right leads. Hence, there exists a terribly weak current for four devices as M_0A_–M_0D_ in the inset of Fig. 4a (for clarity, the diagram of the *I-V* curve has been enlarged under the bias range [0, 1.0 V]). When the applied voltage is larger than 1.2 V, we can find out that the longer the molecular chain is, the current is weaker, implying that the molecular chain could impede the hopping of electrons from the left to right electrodes. The corresponding explanation is displayed in Fig. 5a, letting us concentrate on the transmission peak near the *E*_F_ since the current is largely contributed by the transmission peak [18, 20]. The transmission spectrum of M_0A_ tracks several peaks around the Fermi level; on the contrary, the transmission peak becomes lower and lower from M_0A_ to M_0D_ with the increasing length of the molecular link. For clarity, the inset of Fig. 5a showing the amplifying peak for M_0C_ and M_0D_ refers to account for the reduction of current. Indeed, the pristine AγGYNR is not a perfect electrode to construct a spin (electronic) junction; the issue of N-instituting position is needed to be considered here.

When the devices are doped with single N atom by position 1 (M_1_) or 2 (M_2_), respectively, the opposite situation occurs, and we notice that all the currents are enhanced in Fig. 4b and c. The current obtains a large value under *V* ≤ 1.2 V, and it happens to decrease with the increase of bias for device M_1A_–M_1D_ in Fig. 4b. Note that the obvious NDR behavior can be observed with the dipping of the current occurring between 0.6 and 1.6 V. A similar *I-V* curve displayed that the NDR effect is also found for M_2B_ in Fig. 4c. The maximum of the peak to valley ratio (PVR) can reach up to 5.6. However, the other curves manifest different interesting features originating from the asymmetrical transport pathway in Fig. 3b, which could possibly result in a new physical effect discussed later.

Furthermore, to compare the influence of dopant, we plot the *I-V* curves of M_0_, M_1_, and M_2_ with a four repeated molecular chain in Fig. 4d, indicating that the single N-doping of AγGYNR can effectively enhance the charge transport leading to a strong current. Therefore, the values of the red line (for M_1D_) and the blue line (for M_2D_) are larger than the ones of the black line (for M_0D_). Seen from Fig. 5b, the transport peak of M_1D_ extends to the energy range of − 0.26 eV ≤ *E* ≤ 0.8 eV, contributing to the electron flowing through the central scattering region. There exists a sharp transport peak around the Fermi level for M_2D_ (the blue line) which is little lower than the former one; as a result, a relative weaker current curve appears. Certainly, zero transport gap for M_0D_ (see the black line in Fig. 5b) results in an almost zero value of current. Although there exist many transport peaks at *E* > 1.0 eV, they have tiny contribution for the transport property of device based on AγGYNRs. Hence, single N-doping is an effective method to promote the scattering and hopping of electrons on our designed nanojunctions.

When the pristine devices are doped with double N atoms, the computational results suggest that the total current varies with the substituted positions of dopants for chemical modification. Figure 4e displays that the currents of M_4D_ and M_5D_ are larger than the three ones of M_3D_, M_6D_, and M_7D_. The blue line for M_5D_ exhibits a nearly linear increase as a function of bias voltage with a large current occurring at high bias, while the red one for M_4D_ is a nonlinear curve with a bigger current under the low voltage, because the red transmission peak in Fig. 5c localized around the Fermi level which is easy to be conducted at a lower bias, the blue transmission peak keeps away from the zero energy level which needs a high voltage to breakout the transport channel. So, the current of M_4D_ is larger than the one of M_5D_ at the low bias of [0, 1.2 V], but it begins to become stronger at higher biases.

As explained before, all the transmission spectra of three junctions hold many transmission peaks near the Fermi level (the transmission coefficients are zero at *E*_F_) in Fig. 5d, thereby the low currents produce. Especially, there are many higher peaks of the yellow line at positive energy, supporting that the obvious NDR effect appears. To deeply observe the NDR phenomenon for M_6_, we plot all the *I-V* characteristics from M_6A_ to M_6D_, finding that the NDR effect begins to strengthen with the increase of length for molecular chain. The PVR can increase from 1.7 for M_6A_ to 5.4 for M_6B_, then a PVR maximum of 24.5 can be reached for M_6D_ from the value of 12.8 for M_6C_. Note that the length of the molecular chain can efficiently modulate the occurrence and intensity of NDR behavior.

**Fig. 6:**
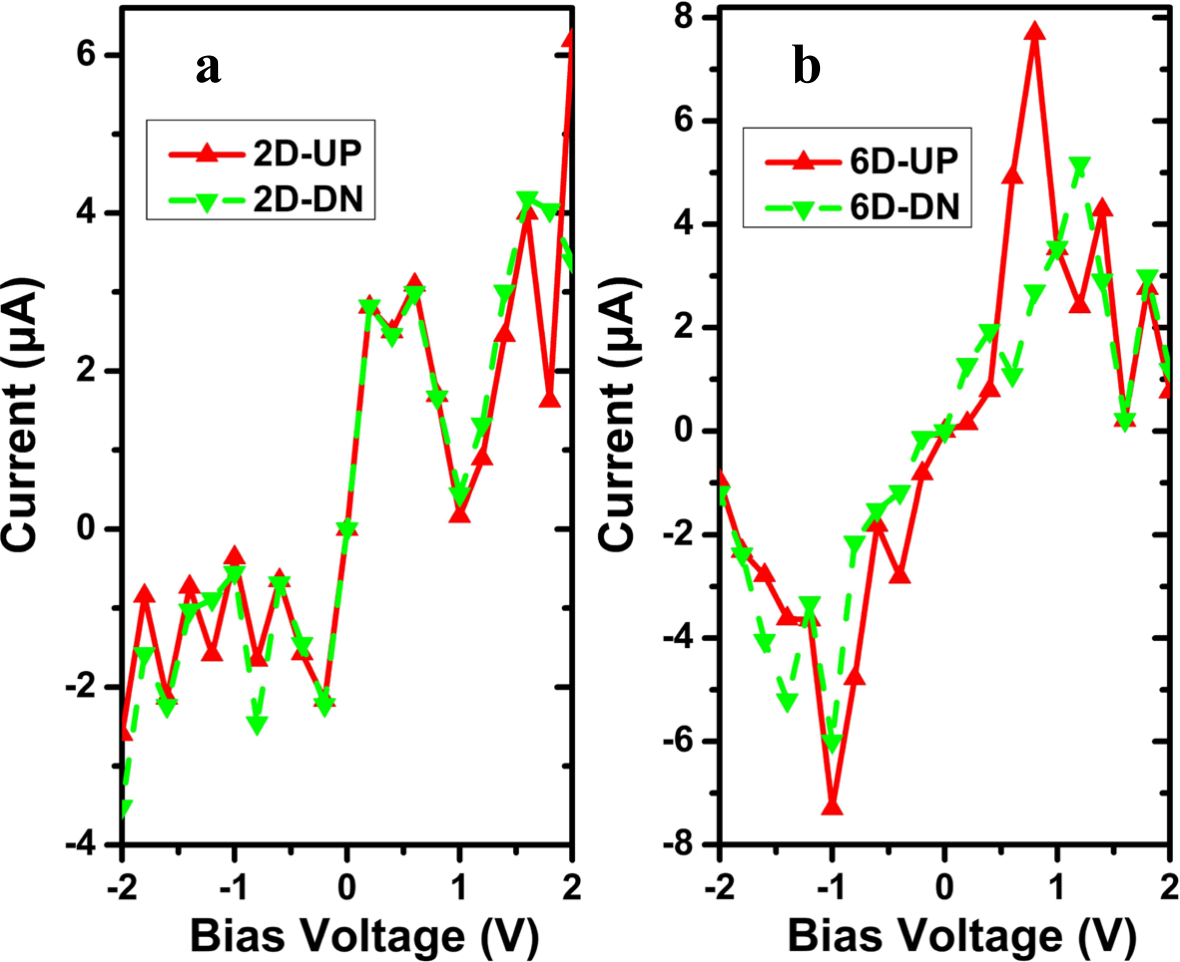
(Color online) The spin-dependent *I-V* curves of AγGYNRs with **a** single N-doping and **b** double N-doping, whose models are shown as M_2D_ and M_6D_ in Fig. 1. All the models only consider the structures considering the molecular chain with four repeated molecular units as D

Meanwhile, the calculated spin-resolved currents as a function of bias voltage are also exhibited for M_2D_ and M_6D_ in Fig. 6, so as to clearly observe the interesting features of spin devices. Within the total bias voltage, both the model devices display visible asymmetric pulse-like *I-V* behavior in Fig. 6 a and b, which yields a perfect NDR phenomenon. The spin-up current for M_2D_ behaves the NDR effect with a PVR of 18.9 in Fig. 6a; nevertheless, the value of PVR reaches up to 36.8 within the spin-up case of M_6D_ between 0.8 and 1.6 V in Fig. 6b and it is also 24 for the spin-down case from 1.2 to 1.6 V. Particularly, for the model 2D in Fig. 6a, the positive currents are stronger than the negative ones at both spin directions, implying that a rectification effect can be found in this device. The rectification ratio (RR) can be defined [70] as the formula: RR(%) = I(V)/│I( − V)│ × 100% for the spin-up (spin-down) current. For the difference of rectification ratio between spin-up and spin-down cases, the RR of spin-up and spin-down current is 480% and 440% at ± 0.6 V, respectively. So, from the viewpoint of practical application, the N-doping not only can impact the band structure [71, 72], but also modulate the device behaviors. The intrinsic physicochemical mechanisms can be used to explain these effects.

**Fig. 7:**
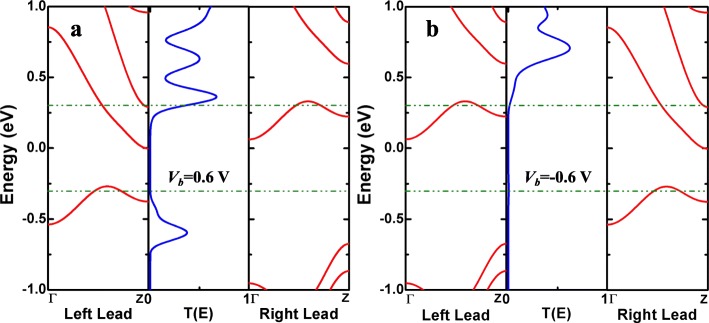
(Color online) The spin-up band structures of the left/right leads and the spin-up transmission spectra of AγGYNRs with single N-doping at the adjoining position for M_2D_ under the biases of **a** 0.6 and **b** − 0.6 V. The region between the double horizontal green dashed lines is the corresponding bias window

To analyze the corresponding mechanisms of the above rectification phenomenon, the spin-dependent band structures at the bias of ± 0.6 V and the transmission spectra of molecular junctions for M_2D_ have been exhibited in Fig. 7. By introducing single N-doping into pristine molecular junction, one can find that the spin-up electronic band of the device at the left electrode shift along the negative energy level, whereas for the right electrode, the band trends to move along the positive direction in Fig. 7a. Whereupon, we can find that the sub-band of the left lead coupling with the one of the right lead at *E* ≈ 0.25 eV and the transmission peak moves into the bias window, resulting in that the transport channel opens at 0.6 V contributing to the charge transport. When a voltage of − 0.6 V is applied for the nanodevice in Fig. 7b, the energy bands of the left and right electrodes move in opposite directions. Although the sub-bands of the left and right electrodes still match each other, there is a nearly zero transmission probability within the bias window, which is the reason of low current at *V*_*b*_ = − 0.6 V. Thereby the rectifying behavior can be obtained here. In general, the phenomenon of rectifier often occurs in the asymmetric molecular structures [20], so the asymmetry of molecular devices is the main reason for the generation of this behavior.

**Fig. 8:**
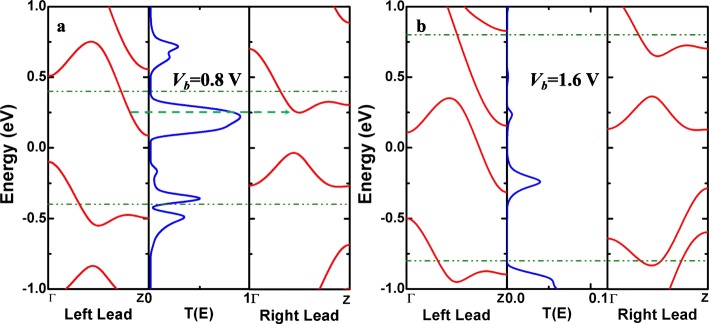
(Color online) The spin-up band structures of the left/right leads and the spin-up transmission spectra of AγGYNRs with double N-doping for M_6D_ under the biases of **a** 0.8 V and **b** 1.6 V. The region between the double horizontal green dashed lines is the corresponding bias window. For clarity, the maximum of transmission spectra in **b** is set to 0.1

There are many NDR effects that have been observed in our proposed models; to better interpret the foundation of NDR, we draw the relative diagrams in Fig. 8. For instance, as expected before, the NDR producing from 0.8 to 1.6 V in a spin-up direction with a high PVR of 36.8 for M_6D_ is chosen as an example here. Under the bias of 0.8 V, the left sub-bands can strongly match with the right ones, the lowest unoccupied molecular orbital (LUMO) behaves a crucial action in Fig. 8a, which results in that a scattering channel can be allowed for spin-up electrons’ hopping. There is a green dashed line with an arrow in Fig. 8a, describing that the transmission channel is open for electron transport at 0.8 V. The highest occupied molecular orbital (HOMO) performing the secondary role also contributes to the electron transport at 0.8 V. When the bias is increased up to 1.6 V, as displayed in Fig. 8b, the energy for the bias window is also expanded to ± 0.8 eV. There happens a lower transmission peak appearing in the corresponding bias window, but weak coupling between the sub-bands of both leads can be found in that energy area, which leads to a terrible weak transmission peak in the scattering area from the left to the right electrode. Hence, the NDR arises in the spin-up current including a high PVR for M_6D_ with the double N-instituting positions. It could be an outstanding candidate for a spin-switch of the nanoelectronic device based on AγGYNRs in the future. Therefore, the generation and transport features of spin-polarized currents are still vital issues for spintronics devices [73].

## Conclusions

In summary, the comprehensive ab initio calculations based on the density functional theory combined with non-equilibrium Green’s function formalism on the 2D armchair 3-γ-graphyne sheets and nanoribbons with the incorporation of nitrogen atoms possess many electronic and transport characteristics that are obviously different from those of well-known graphene and typical graphynes. By exploring the impact of single or double N-doping defects of AγGYNRs, our results confirm that band structures of super unit cells are highly dependent on the positions of the dopant in the central C_6_ ring of nanoribbons. We can obtain some semiconducting nanoribbons with narrow band gap or conductors of AγGYNRs. With regard to the transport properties, the different lengths of molecular chains induce interesting negative difference resistance behavior which has been expected for nanoelectronic junctions. In particular, the hexagonal rings in the middle of nanoribbons hold a vital role in the transport properties. The longer the molecular chain is, the more obvious NDR effect can be observed in the junctions including N-instituting positions. For the crucial N-doping for junctions M_2D_ and M_6D_, the spin-polarized currents with the maximums of rectification ratio and peak to valley ratio of 480% and 36.8 in spin-up direction have been found, respectively. We propose the distinct physical mechanisms notably suggesting that the molecular junctions of AγGYNRs endow potential applications for future nanoelectronic devices.

## Data Availability

The design of nanojunctions and computational calculations were carried out by ATK.

## Abbreviations

2D: Two-dimensional
ATK: Atomistix ToolKit
AγGYNR: Armchair-edged γ-graphyne nanoribbon
C_6_: Six-membered carbon
DN: Spin-down
*E*_F_: Fermi energy
HOMO: Highest occupied molecular orbital
LUMO: Lowest unoccupied molecular orbital
NDR: Negative difference resistance
PDOS: Projected density of state
PVR: Peak to valley ratio
RR: Rectification ratio
UP: Spin-up
